# Broken Bone-Shafts

**Published:** 1896-10

**Authors:** Thos. H. Manley

**Affiliations:** New York


### BROKEN BONE-SHAFTS.

By THOS. H. MANLEY, M.D.,
New York.

Medical Journals must be filled with something, no matter how absurd or preposterous the subject may be, provided only that it be sustained by the force of enough assurance.

This is about the position of fracture-treatment at the present time. For instance, M. Lucas-Champiouniere, of Paris, in his late work on fractures, condemns the fracture-box and the splint, and advises the man with a broken leg to commence the ambulant course and move about the day after the injury. But, is not the mangled member to be placed in some sort of brace, and it with the body placed at rest, for at least a few days?

Bless your innocent soul, no!

All that belongs to an antiquated age and has no place in modern fracture-therapy. And we, gullible Americans, at one gulp, swallow all this stuff, simply because it is of European importation. During my own experience in the treatment of a great number of traumatisms—more than five thousand fractures having come under my observation while serving my interneship in 1875 and 1876—the older methods of fracture-treatment were in full force; and it is my conviction to-day, that simple fractures then were treated with much greater success than they are to-day. We had then what we knew as the "English" method and the "French." The former permitted a mangled limb to remain at rest until intumescence and primary inflammation disappeared, before a rigid adjustment was applied, and required muscular relaxation and joint flexure in posture. The latter called for immediate osseous rectification and permanent immobilization in the extended position; a very seductive thing, in theory.

The improvement in the treatment of compound comminuted fractures in the past twenty years has been enormous. Sir Joseph Lister's work opened the way to a revolution in this direction; and Ollier has placed the profession under an eternal obligation to him for teaching us the art of osteoplasty, in every description of bone injury.

In consequence of these advances on conservative lines at the present time, in no description of fracture whatever, in which the limb is not totally destroyed, no thought of amputation should be entertained until all the resources of our art have been exhausted to save the limb.

Fracture occurs most commonly in children; next, in the male sex, until the meridian of life is past, and after this, as Duplay shows, as degenerative changes are more rapid in the female, fracture with her is most common. This is a class always difficult to treat. Confinement to bed kills many; especially the very fat, or the feeble. In this class the earlier we can get them out the better.

Let it be remembered that fractures, like a division through the soft parts, unite, or fuse, by two distinct processes—viz., primary or secondary union. Many times have I seen simple fracture with no displacement solidly unite in a week. On these no splints were applied. The knees were flexed and the leg comfortably placed on a straw pillow.

We can never entirely dispense with splints, but there can be no doubt, as commonly used, they do more harm than good.

Of all things we must guard the fullest liberty of the circulation. Tight splints sometimes seriously menace it. A year ago I made an extensive study of the effect of fracture on the circulation; using the transparent webbing of the frog's foot for the purpose. I found, when I fractured the femur, the capillary circulation was immediately arrested by injury of the femoral artery. After the circulation was re-established splints were applied to the limb. These arrested the circulation in whole capillary territories, and greatly moderated the velocity of the blood-tide in the arterioles.

In some cases of non-union of a bone-shaft, more perfect fixation (?) by secure splinting may be tried; of course, in vain; when the experienced surgeon takes the case in hand, he removes every description of dressing, and orders free joint-action on either end of the fracture.

Massage, baths, and electricity utilized, the circulation is liberated and stimulated, the absorbents are called into fresh activity, edema disappears, and osseous consolidation soon welds the loose fragments solidly together.

At the elbow in the child, the ankle in the male adult, and the wrist in the middle-aged adult, or elderly persons, mostly women, fractures often occur. In spite of any special plan of treatment adopted, as a rule they leave a certain degree of impairment in function, and more or less deformity.

As a matter of fact all the above group of complicated cases belong to this class. Muscular relaxation, and the fullest possible freedom of the circulation are the *sine qua non* in their treatment.

Much difference of opinion yet remains on the period when passive motion should begin. Professor Lewis A. Stimpson and other distinguished authorities on fracture are rather inclined not to employ this indiscriminately in their cases.

In conclusion, let it never be forgotten that, in those traumas attended with disorganization of bone, the osseous fracture is but one lesion; for the nerve-trunks, blood-vessels, and muscles all share in the mutilation of tissue, and demand equally with the bone our closest attention. Dr. Andrew J. McCosh, of New York, in a recent excellent summary on the "Year's Progress in Surgery," very judiciously advises, that the general practitioner will do well, in the treatment of fractures, not to prematurely embrace the latest doctrines on their therapy; nor any method which has not the support of the majority of surgeons. In any event, in every description of severe fracture, the physician hardly does full justice to his patient if he neglects to take in one or more consultants. In the city, among the poor, this is obviated by sending severe cases to the hospital.

---

### CYCLING.

As preventive measures against the dangers of this fascinating sport may be mentioned: First, the use of a low gear; second, the upright position in riding; third, adequate food when riding and the avoidance of muscle poisons, such as beef tea; fourth, the avoidance of preparations of kola and coca, which numb the sense of weariness; and fifth, on no account should the cyclist continue riding after he has commenced to feel short of breath, or when there is the slightest sense of weariness in the chest.—Dr. Herschell in *Lancet*.

